# Cardiac volumes can be quantified accurately during free-breathing in young patients with congenital heart disease by cardiovascular magnetic resonance

**DOI:** 10.1186/1532-429X-16-S1-P124

**Published:** 2014-01-16

**Authors:** Ahmed E Kharabish, Naira Mkrtchyan, Christian Meierhofer, Stefan Martinoff, Peter Ewert, Heiko Stern, Sohrab Fratz

**Affiliations:** 1 Pediatric Cardiology and Congenital Heart Defects, German Heart Center, Munich, German; 2 Radiology and Nuclear Medicine, German Heart Center, Munich, Germany

## Background

Cardiovascular Magnetic Resonance (CMR) with respiratory commands is the gold standard technique to measure cardiac volumes. Although cardiac volumes can be measured during free breathing in patients with congenital heart disease (CHD), its accuracy is unknown. Therefore, the aim of this study was to compare cardiac volumes acquired during free breathing with volumes acquired during breath hold commands.

## Methods

Cardiac volumes were measured in every patient using both free breathing and breath hold techniques, by a routine standard steady state free precession (SSFP) cine sequence in axial slice orientation. The sequence parameters were always identical besides the number of averages being three during free breathing and one during breath holds. Volumes were determined in a blinded fashion by endocardial contouring and then correlated using the coefficient of determination and compared by Bland Altman analysis. Eleven younger patients with CHD aged median 4 yrs, range 3 months-14 yrs, were examined under general anesthesia and intubation (intubated younger patient group). Twelve older patients with CHD aged median 20 yrs, range 11-61 yrs were examined consciously (conscious older patient group).

## Results

**Figure 1:**
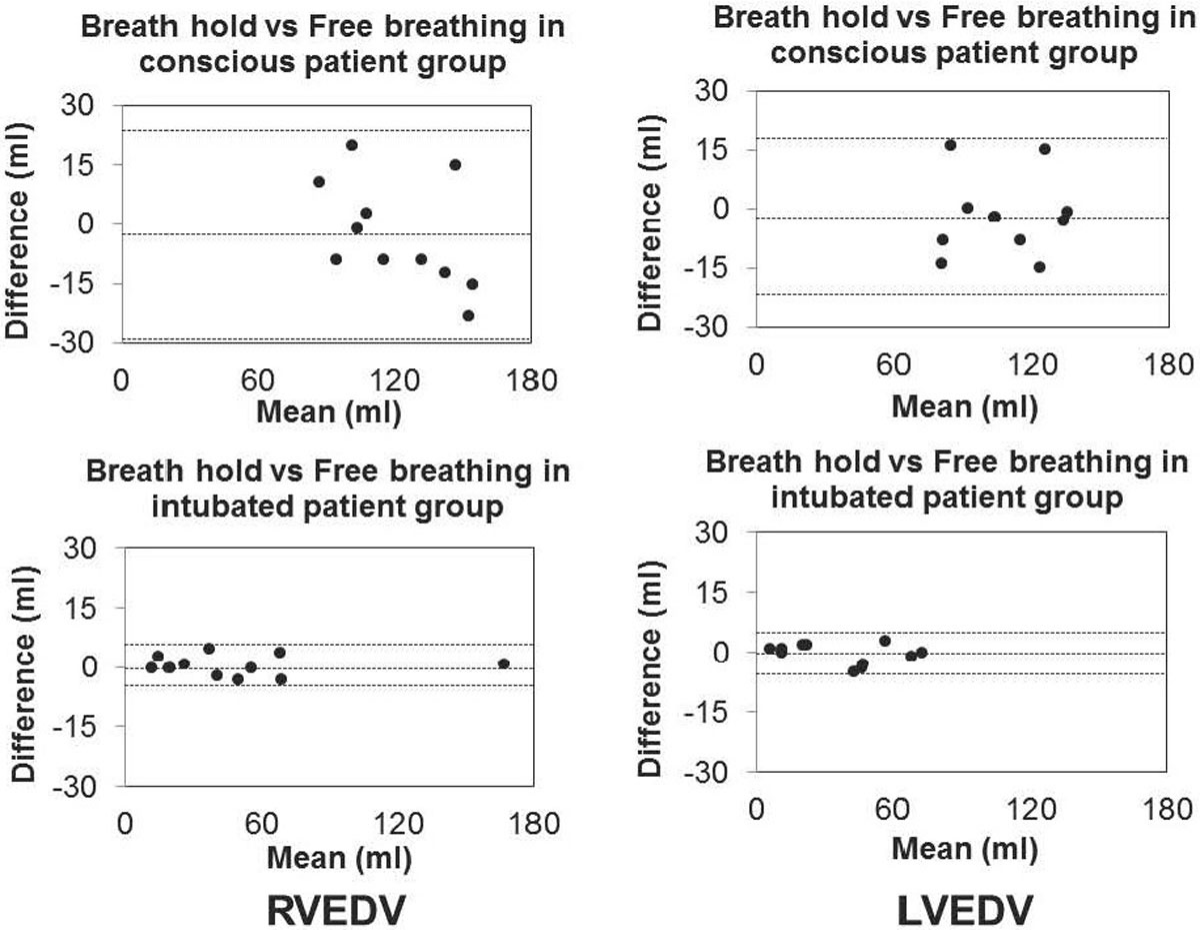
**Bland Altman graphs showing differences of means between the breath hold and free breathing methods on the y-axis and the mean of both methods on the x-axis in ml in the right ventricular end diastolic volume (RVEDV) and left ventricular end diastolic volume (LVEDV)**. In the consciously examined group the upper and lower limits of agreement of LVEDV: 17.9 ml and -21.6 ml, RVEDV: 23.8 ml and -29 ml. In the intubated examined group the upper and lower of agreement of LVEDV: 4.7 ml and -5.5 ml, RVEDV: 5.7 ml and -4.6 ml.

The agreement of the end systolic volume (ESV) and end diastolic volumes (EDV) between scanning with and without respiratory commands in both ventricles was excellent in the intubated younger patients (LVEDV: r2 = 0.98, LVESV: r2 = 0.99, RVEDV: r2 = 0.99, RVESV: r2 = 0.99) and less acceptable in the consciously examined older patients (LVESV: r2 = 0.87, LVEDV: r2 = 0.78, RVESV: r2 = 0.84 and RVEDV: r2 = 0.84) (see Figure 1).

## Conclusions

Cardiac volumes can be quantified very accurately during free-breathing in young patients with congenital heart disease by CMR using standard routine imaging techniques. If needed, even in older patients cardiac volumes can be quantified reasonably accurate during free-breathing. Therefore, free-breathing is an alternative technique for patients not able to hold their breath and thus obliterating the need of anesthesia for many patients undergoing CMR. In addition it will provide a chance for the CMR to assess the hemodynamics in a resting physiologic condition.

## Funding

All authors have no conflict of interest.

